# Does the Interdependence between Oxidative Stress and Inflammation Explain the Antioxidant Paradox?

**DOI:** 10.1155/2016/5698931

**Published:** 2016-01-05

**Authors:** Subrata Kumar Biswas

**Affiliations:** Department of Biochemistry, Bangabandhu Sheikh Mujib Medical University (BSMMU), Shahbag, Dhaka 1000, Bangladesh

## Abstract

Oxidative stress has been implicated in many chronic diseases. However, antioxidant trials are so far largely unsuccessful as a preventive or curative measure. Chronic low-grade inflammatory process, on the other hand, plays a central role in the pathogenesis of a number of chronic diseases. Oxidative stress and inflammation are closely related pathophysiological processes, one of which can be easily induced by another. Thus, both processes are simultaneously found in many pathological conditions. Therefore, the failure of antioxidant trials might result from failure to select appropriate agents that specifically target both inflammation and oxidative stress or failure to use both antioxidants and anti-inflammatory agents simultaneously or use of nonselective agents that block some of the oxidative and/or inflammatory pathways but exaggerate the others. To examine whether the interdependence between oxidative stress and inflammation can explain the antioxidant paradox we discussed in the present review the basic aspects of oxidative stress and inflammation and their relationship and dependence.

## 1. Introduction

Inflammation and oxidative stress are linked with a number of chronic diseases including diabetes and diabetic complications, hypertension and cardiovascular diseases, neurodegenerative diseases, alcoholic liver disease, chronic kidney disease, cancer, and aging [1–8]. There is no doubt that the chronic low-grade inflammatory process plays a central role in the pathogenesis of many chronic diseases [9]. However, epidemiological and experimental studies strongly suggest a contribution of oxidative stress in many human diseases [1, 2]. A vast number of researchers all over the world have investigated whether antioxidants are capable of preventing diseases like cardiovascular diseases, cancer, diabetic complications, Alzheimer's disease, and so forth [10–12]. However, results of these antioxidant trials are largely frustrating in human patients; although some of the trials show beneficial health effects, others show either no effect or even harmful effects [10–12]. Thus, the findings of antioxidant trials have raised a great deal of uncertainty about the role of oxidative stress in the pathogenesis of human diseases. Although a number of explanations have been proposed to clarify this discrepancy between findings of clinical trials and those of epidemiological/experimental studies, it has appeared as a new puzzle in medical science and is known as antioxidant paradox [13, 14].

Inflammatory cells liberate a number of reactive species at the site of inflammation leading to exaggerated oxidative stress [9]. On the other hand, a number of reactive oxygen/nitrogen species can initiate intracellular signaling cascade that enhances proinflammatory gene expression [15, 16]. Thus, inflammation and oxidative stress are closely related pathophysiological events that are tightly linked with one another. In fact, experimental data show the simultaneous existence of low-grade chronic inflammation and oxidative stress in many chronic diseases like diabetic complications, cardiovascular and neurodegenerative diseases, alcoholic liver disease, and chronic kidney disease [1–8, 17–19]. Therefore, failure of selection of agents that specifically target both inflammation and oxidative stress or failure to use both antioxidant and anti-inflammatory agents simultaneously or use of nonselective agents that block some of the oxidative and/or inflammatory pathways but exaggerate the others might be responsible for the failures of the antioxidant clinical trials. This idea tempted us to review basic aspects of oxidative stress and inflammation and their relationship and dependence.

## 2. Oxidative Stress and Inflammation: Basic Aspects

### 2.1. Prooxidant, Antioxidant, and Oxidative Stress

Chemically,* oxidation* is defined as the removal of electrons and* reduction* as the gain of electrons [20]. The general meaning of the term* oxidant* is “oxidizing agent.” In reactions, a free radical may act as an oxidizing agent by taking a single electron from other species or as a reducing agent by donating a single electron to other species [21]. The term* prooxidant* is not well defined; it is generally considered that a prooxidant is any substance that can generate reactive species or capable of inducing oxidative stress. However, an* antioxidant* is defined as any substance that when present at low concentrations compared with those of an oxidizable substrate significantly delays or prevents oxidation of that substrate [22].* Oxidative stress* is conventionally defined as an imbalance between prooxidant stress and antioxidant defense. However, recent evidence indicates that the disruption of redox signaling is an important aspect of oxidative stress, sometimes more important than the prooxidant-antioxidant imbalance or the tissue damage induced by such imbalance [23]. Therefore a new definition of oxidative stress has been proposed as “an imbalance between oxidants and antioxidants in favor of the oxidants, leading to a disruption of redox signaling and control and/or molecular damage” [23]. Consequences of oxidative stress can be very subtle to very serious (including oxidative damage to biomolecules, disruption of signal transduction, mutation, and cell death) depending upon the balance between reactive species generation and the antioxidant defense [22].

### 2.2. Free Radical

A* free radical* is any species that contains one or more unpaired electrons, that is, electrons singly occupying an atomic or molecular orbital [22]. Because electrons are more stable when paired together in orbitals, free radicals are generally reactive with other species [24]. Unpaired electrons have a strong tendency to form pair to become stable. Therefore, a radical might donate its unpaired electron to another molecule or it might steal an electron from another molecule in order to form a pair. However, if a radical gives one electron to another molecule or takes one from another molecule, that other molecule itself becomes a radical. Thus an important feature of free radical mediated reactions is that they tend to proceed as chain reaction [24].

### 2.3. Reactive Species

There are three different classes of* reactive species* relevant in biology and medicine. They are (a) reactive oxygen species (ROS), (b) reactive nitrogen species, and (c) reactive chlorine species. A reactive species may be a free radical or a nonradical in structure [22]. ROS is a collective term including both oxygen radicals and certain nonradicals that either are oxidizing agents or are easily converted into radicals or both. Superoxide (O_2_^•−^) and hydrogen peroxide (H_2_O_2_) are examples of radical and nonradical ROS, respectively. Similarly, reactive nitrogen species is a collective term including radicals (nitric oxide, NO^•^) and nonradicals (peroxynitrite, ONOO^−^), and reactive chlorine species is also a collective term including radicals (atomic chlorine, Cl^•^) and nonradicals (hypochlorous acid, HOCl) [21]. A list of important reactive species in biological system is shown in Table 1, as reviewed in [21].

Among the reactive species, the free radical superoxide anion (O_2_^•−^) is of critical importance, because O_2_^•−^ is the primary species produced in the cells, and many other reactive species of physiological significance, including H_2_O_2_, hydroxyl radical (OH^•^), and ONOO^−^, are derived from O_2_^•−^ as products of the downstream reaction cascade [25]. The endogenous sources of O_2_^•−^ in mammals include NADPH oxidases, the mitochondrial electron-transport chain, xanthine oxidases, cyclooxygenases and lipoxygenases, nitric oxide synthases, and cytochrome P450s [26–31].

### 2.4. Oxidative Tissue Injury

There are many pathways for inducing ROS-mediated oxidative damage to biomolecules. One such pathway starts from the interaction between two commonly found free radicals* in vivo*, O_2_^•−^ and NO^•^:

(1) $$\begin{array}{c} {O_{2}}^{•-}+{\text{NO}}^{•}⟶{\text{ONOO}}^{-}\left(\;\text{Peroxynitrite}\;\right) \end{array}$$

The reaction product is peroxynitrite, which, at physiological pH, rapidly protonates to peroxynitrous acid (ONOOH). This powerful oxidizing and nitrating agent has the capacity to damage proteins, lipids, and DNA [21].

Nitration of tyrosine residues of proteins generates nitrotyrosine which is widely used as a biomarker for oxidative and nitrosative stress. But the nitrotyrosine is not a specific biomarker for peroxynitrite formation because there are several other nitrating agents* in vivo* [32]. However, nitration of proteins is very dangerous for the cell or the organism. Nitration of structural proteins, including neurofilaments and actin, can disrupt filament assembly with major pathological consequences [33]. On the other hand, nitration of signaling molecules or transcription factors can greatly modify the physiological function of the affected proteins [34]. Furthermore, peroxynitrite mediates calcium-dependent mitochondrial dysfunction and cell death via activation of calpains [35].

Hydroxyl free radical (OH^•^) induced lipid peroxidation and DNA hydroxylation are also major pathways for oxidative damage. The OH^•^ radical is the most reactive species known to chemistry as it can attack and damage almost every molecule found in living cells [24]. The OH^•^ radical can react with the ring structure of guanine in DNA forming the adduct 8-hydroxy-2′-deoxyguanosine (8-OHdG) radical which can propagate a chain reaction through the DNA and cause chemical alteration of the bases as well as DNA strand breakage. Imperfect repair of such DNA damage can lead to mutations, arrest of cell growth, or apoptosis [36]. The OH^•^ radical can also initiate chain reaction by reacting with membrane lipids leading to lipid peroxidation. The overall effects of lipid peroxidation are to decrease membrane fluidity, increase the leakiness of the membrane, and damage membrane proteins, thereby inactivating receptors, enzymes, and ion channels [21].

### 2.5. Antioxidant System

**Figure 1:**
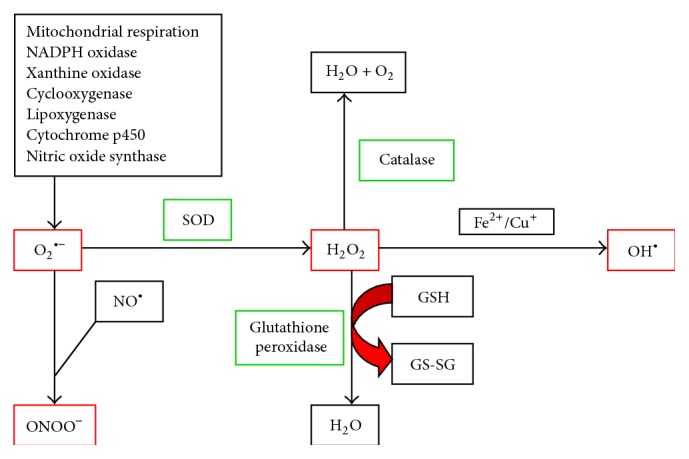
Major prooxidant-antioxidant reactions relevant in biological system. Superoxide (O_2_^•−^) produced from a number of sources acts as a primary reactive species. O_2_^•−^ rapidly reacts with nitric oxide (NO^•^) to produce peroxynitrite (ONOO^−^) or is catalyzed by superoxide dismutase (SOD) to produce hydrogen peroxide (H_2_O_2_). H_2_O_2_ can be neutralized by catalase or glutathione peroxidase. However, in presence of transition metal ions, like iron (Fe^2+^) and copper (Cu^+^), highly toxic hydroxyl free radicals (OH^•^) can be produced from H_2_O_2_ via the Fenton reaction. Reactive species are shown in red and antioxidant enzymes are shown in green boxes. GSH, reduced glutathione; GS-SG, oxidized glutathione.

To minimize the oxidative damage, antioxidant systems have been evolved. Enzymatic antioxidants like superoxide dismutase (SOD), catalase, and glutathione peroxidase and nonenzymatic antioxidants like vitamins C and E, glutathione (reduced form, GSH), and beta-carotene provide major protection against oxidative stress by neutralizing or scavenging reactive species or by breaking the chain reactions, as reviewed in [37]. In addition, transferrin, ceruloplasmin, and albumin also play antioxidant role by sequestering transition metal ions, like iron and copper, as the metal ions rapidly react with H_2_O_2_ to yield highly toxic hydroxyl radical (OH^•^) by Fenton reaction [24]. Major prooxidant-antioxidant reactions are summarized in Figure 1 [17, 21, 26, 38].

However, reactive species are not always harmful. They help phagocytes to kill microorganisms and modulate signaling events by redox (reduction and oxidation) regulation and thereby affect the phosphorylation and dephosphorylation of enzymes and transcription factors [21]. In fact, in his recent hypothesis Watson postulated that diabetes, dementias, cardiovascular diseases, and some cancers are accelerated or even caused by failure to generate sufficient ROS [39]. In support of this hypothesis recent studies showed that insufficient levels of ROS, due to failure to induce apoptosis, promote survival of malignant cells and thereby contribute to unabated growth of tumors [40, 41]. Thus ROS produce obvious beneficial health effects at least in some situations. Similarly, the antioxidants may also be good or bad for health depending on the situation. For example, in premalignant stage the antioxidants are good as they can inhibit ROS-induced DNA damage and malignant transformation of cells exposed to carcinogens, like arsenic and cadmium, through alleviating ROS [42, 43]. However, in transformed cells or in cancer cells, antioxidants are bad as they can decrease ROS and thereby can inhibit ROS-induced apoptosis of genetically damaged cells leading to increased cell survival, proliferation, and carcinogenesis [40, 41]. Thus the antioxidants may exert either beneficial or harmful effects depending on the cellular requirement for ROS at a particular situation.

### 2.6. Inflammation

Inflammation is commonly considered as a complex reaction in the vascularized connective tissue in response to exogenous and endogenous stimuli. The ultimate goal of this protective response is to rid the organism of both the initial cause of cell injury and the consequences of such injury. However, exaggerated or unregulated prolonged inflammatory process can induce tissue damage and is the cause for many chronic diseases [9]. A critical component of inflammation is the infiltration of inflammatory cells, like neutrophils, monocytes, and lymphocytes, to the site of stimulus. The infiltration of leukocytes to the site of inflammation is a highly coordinated process involving margination, rolling, and adhesion of leukocytes to the vascular endothelium, transmigration across the endothelium, and migration toward a chemotactic stimulus. The participation of a number of adhesion molecules, including selectins, intercellular adhesion molecule-1 (ICAM-1), and vascular cell adhesion molecule-1 (VCAM-1), and their respective leukocyte receptors and chemokines like monocyte chemoattractant protein 1 (MCP-1) or interleukin-8 (IL-8) is crucial for the inflammatory cellular infiltration [9]. At the site of inflammation the activated inflammatory cells release many enzymes (neutral proteases, elastase, collagenase, acid hydrolases, phosphatases, lipases, etc.), reactive species (superoxide, hydrogen peroxide, hydroxyl radical, hypochlorous acid, etc.), and chemical mediators (eicosanoids, complement components, cytokines, chemokines, nitric oxide, etc.) and thereby induce tissue damage and oxidative stress [9].

### 2.7. Pattern Recognition Receptors

At the onset of inflammation the infection or tissue damage is sensed by the pattern recognition receptors like Toll-like receptors (TLR), NOD-like receptors (NLR), and the receptor for advanced glycation end products (RAGE). These receptors are activated upon binding with the molecules known as pathogen activated molecular patterns and damage activated molecular patterns [44, 45]. Upon activation the pattern recognition receptors engage in signal transduction pathways that activate transcription factors such as nuclear factor-*κ*B (NF-*κ*B) and activating protein-1 (AP-1). These factors act in combinatorial and cell-specific manner to induce proinflammatory gene expression, exert antimicrobial functions, and recruit additional immune cells [44, 45]. However, recent findings implicate that costimulation of TLR produces oxidative stress with unbalance of proinflammatory and anti-inflammatory cytokine production [46]. Furthermore, activation of RAGE by binding with its ligands (advanced glycation end products, S100/calgranulins, and high mobility group box 1) can produce sustained inflammation and oxidative stress [45, 47].

### 2.8. Nuclear Factor-*κ*B

The nuclear factor-*κ*B (NF-*κ*B) is a transcription factor of major importance in inflammation, stress response, cell differentiation, or proliferation as well as cell death. The NF-*κ*B regulates the gene expression of proinflammatory cytokines, chemokines, inflammatory enzymes, adhesion molecules, receptors, and microRNA [48, 49]. The NF-*κ*B/Rel family includes NF-*κ*B1 (p50/p105), NF-*κ*B2 (p52/p100), p65, RelB, and cRel. Most members of this family form dimers with each other, the heterodimer consisting of p50 and p65 subunits being the most prevalent activated form of NF-*κ*B [50]. In resting cells, NF-*κ*B dimers remain in the cytoplasm as an inactive form bound to the inhibitory protein I*κ*B. Upon cellular activation by extracellular stimuli, I*κ*B is phosphorylated, ubiquitinylated, and ultimately degraded by the proteasome system. As a result, NF-*κ*B dimers are translocated into the nucleus and activate the transcription of target genes [51]. The transcription factor NF-*κ*B can be activated by a number of different stimuli, including bacterial lipopolysaccharides, viral agents, phytohemagglutinin, cytokines (tumor necrosis factor-*α* and IL), and protein kinase C activators (phorbol esters) [48]. Importantly, oxidative stress or intracellular redox status is also involved in the activation of NF-*κ*B; particularly, H_2_O_2_ has been found to activate NF-*κ*B and antioxidants have been demonstrated to block NF-*κ*B activation [15, 16]. However, this basic concept of the activation and function of NF-*κ*B system is obviously incomplete and too simple because the expression of the genes that mediate inflammatory process is not the only effect of the NF-*κ*B activation. It has been shown that the NF-*κ*B subunits also contribute to orchestrated gene clusters required for the resolution of inflammation and to alleviation of oxidative stress by increased expression of antioxidant enzymes (MnSOD) [52–54]. How the NF-*κ*B system regulates the expression of apparently conflicting genes in health and disease is quite complicated and has not been fully clear yet. However, it is generally considered that the NF-*κ*B system is regulated in a cell- and stimulus-specific manner producing a diverse spectrum of effects [55].

## 3. Inflammation and Oxidative Stress: Relationship and Dependence

Numerous studies support an interdependent relationship between inflammation and oxidative stress, as reviewed in [56, 57]. During inflammatory process the activated phagocytic cells like neutrophils and macrophages produce large amounts of ROS and reactive nitrogen and chlorine species including superoxide, hydrogen peroxide, hydroxyl free radical, nitric oxide, peroxynitrite, and hypochlorous acid to kill the invading agents [58]. Under pathological inflammatory conditions there may be exaggerated generation of reactive species and some of those reactive species diffuse out of the phagocytic cells and thus they can induce localized oxidative stress and tissue injury [58]. However, apart from the direct production of reactive species by the professional phagocytic cells, the nonphagocytic cells can also produce reactive species in response to proinflammatory cytokines [59, 60]. The proinflammatory cytokine interferon-*γ* and the proinflammatory component of bacterial cell wall lipopolysaccharide have been found to synergistically increase ROS production in human pancreatic cancer cell lines and in human pancreatitis through TLR-4-NF-*κ*B-dependent expression of Duox2, a member of NADPH oxidase family [59]. Recent finding also showed that the costimulation of TLR produces oxidative stress with unbalance of proinflammatory and anti-inflammatory cytokine production, as mentioned above [46]. Furthermore, the inflammatory cytokine IL-6 has been found to produce ROS through increased expression of NADPH oxidase 4 (NOX4) in non-small cell lung cancer [60]. The NOX4 overexpression has also been found to enhance IL-6 production, and a positive reciprocal feedback loop has been found between IL-6 and NOX4, the two mediators of inflammation and oxidative stress, respectively [60].

As the inflammatory process can induce oxidative stress, the oxidative stress can also induce inflammation through activation of multiple pathways. The reactive species hydrogen peroxide can induce inflammation through activation of transcription factor NF-*κ*B, as mentioned above [15, 16]. Furthermore, oxidative stress plays an important role in the activation of NOD-like receptor protein 3 (NLRP3) inflammasome [61–63]. The NLRP3 inflammasome is an oligomeric molecular complex that triggers innate immune defenses through the maturation of proinflammatory cytokines like IL-1*β* and IL-18 [64]. Several mechanisms of ROS-mediated activation of NLRP3 inflammasome have recently been shown [61–63]. The ROS released from damaged mitochondria has been shown to activate NLRP3 inflammasomes leading to IL-1*β* secretion and localized inflammation [61]. Oxidized mitochondrial DNA has also been found to activate NLRP3 inflammasomes during apoptosis [62]. Furthermore, in conditions of oxidative stress the ROS causes the thioredoxin-interacting protein, an inhibitor of endogenous antioxidant thioredoxin, to dissociate from thioredoxin and to bind with NLRP3 leading to activation of NLRP3 inflammasome [63].

The ROS-induced DNA base modification has also been shown to induce inflammation. The base excision repair of oxidatively damaged/modified DNA base (7,8-dihydro-8-oxoguanine) by 8-oxoguanine-DNA glyoxalase-1 induces a signaling cascade that culminates in the activation of NF-*κ*B pathway resulting in proinflammatory gene expression and inflammatory cell accumulation [65]. The 8-isoprostane, an end product of arachidonic acid belonging to the F2-isoprostanes and a marker of oxidative stress, has been found to increase the expression of inflammatory chemokine IL-8 in human macrophages through activation of mitogen-activated protein kinases (MAP kinases) [66]. Furthermore, the oxidative stress induced oxidation of the extracellular redox potential of plasma cysteine (Cys) and its disulfide cystine (CySS) has been shown to trigger monocyte adhesion to vascular endothelial cells, activate NF-*κ*B, and increase the expression of proinflammatory cytokine IL-1*β* [67, 68].

**Figure 2:**
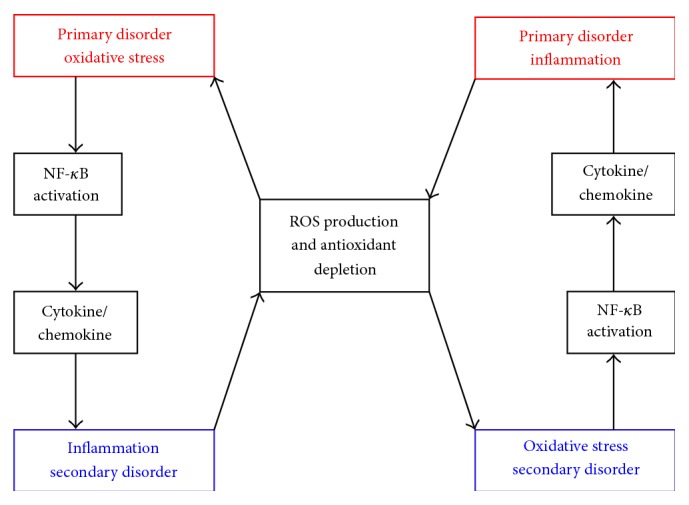
Overview of interdependence between oxidative stress and inflammation. When oxidative stress appears as a primary disorder inflammation develops as a secondary disorder and further enhances oxidative stress. On the other hand, inflammation as a primary disorder can induce oxidative stress as a secondary disorder which can further enhance inflammation. NF-*κ*B, nuclear factor-*κ*B; ROS, reactive oxygen species.

The above discussion indicates that the inflammation and oxidative stress are closely related and tightly linked interdependent pathophysiological processes.  Figure 2 depicts this close and interdependent relationship between oxidative stress and inflammation, although the sequence of events is not so simple. Many other redox-sensitive signal transduction pathways like c-Jun N-terminal kinase (JNK) and p38 MAP kinase and transcription factor AP-1 also participate to set up a vicious cycle between inflammation and oxidative stress [69]. If oxidative stress appears as the primary abnormality in an organ, inflammation will eventually develop and will further accentuate oxidative stress. Conversely, if inflammation is the primary event, oxidative stress will develop as a consequence which will further exaggerate inflammation [69]. Therefore, identification of primary abnormality could be of great clinical importance, as the treatment of the primary disorder is likely to ensure a sustained relief from the problem.

The identification of primary abnormality is however not easy because the oxidative stress and inflammation are tightly linked and are interdependent pathophysiological events. Like many other chronic diseases, oxidative stress and inflammation in the kidney are a common finding in spontaneously hypertensive rats (SHR) and in different other models of hereditary and acquired hypertension [69, 70]. Moreover, the finding that the inflammation and oxidative stress appear in the kidney before development of hypertension in SHR suggested that those renal abnormalities could be causally linked to hypertension [71–73]. In an attempt to identify primary defect, an important question—which one appears first between oxidative stress and inflammation in the kidney in SHR—was investigated by our group [3]. To answer this question, 2-week- and 3-week-old prehypertensive SHR and age-matched WKY rats were studied, and a clear elevation of both renal inflammation and oxidative stress was found in the SHR at 3-week time point. However, at 2 weeks, although the proinflammatory markers were not found to be elevated some of the prooxidant and antioxidant markers were found to be elevated in the kidney in SHR suggesting a possible early disruption of redox balance [3]. To make sure that oxidative stress appears before inflammation in the kidney the 2-week-old SHR were treated with antioxidants for one week. The antioxidant therapy reduced renal oxidative stress which was associated with significant reduction of tubulointerstitial macrophage infiltration in the renal cortex [3]. This finding suggested that oxidative stress, but not inflammation, is the primary defect in the kidney in SHR. In line with this finding several studies also found beneficial effects of antioxidant therapy on blood pressure, renal inflammation, and oxidative stress in animal models of hypertension [74–76]. However, it is not known whether renal oxidative stress also appears as a primary defect in prehypertensive human subjects. Because such studies are difficult to conduct in humans the findings of those animal studies are still of limited clinical significance.

## 4. Antioxidant Paradox: Probable Explanations

The exact reason for the failure of antioxidants to produce beneficial effects in human diseases that have been linked with oxidative stress is not yet clear; however, several explanations have been proposed [13, 14, 77]. One theoretical explanation is that the association of oxidative stress to different human diseases is probably not causative in many of the cases, if not all, and so the antioxidants are ineffective [14, 77]. This argument is confusing since there is a vast body of literature showing a contribution of oxidative stress to diseases like cancer and neurodegenerative disease [14]. Another explanation is that the type and dosage of antioxidants used in clinical trials perhaps had not alleviated the oxidative stress in a tissue- or cell-specific manner (i.e., on target) and therefore did not produce any effect or produced harmful effects [13, 14, 77]. This explanation may be considered valid since the antioxidant network is complex and interrelated. For example, SOD can catalyze O_2_^•−^ but it in turn produces another ROS, H_2_O_2_, as a product [21]; the antioxidant vitamin E acts in the membrane while vitamin C acts in the extracellular and intracellular aqueous media; and both higher and lower intakes of vitamin C, compared with recommended daily allowance, are associated with free radical damage to DNA [78–80]. Furthermore, failure of antioxidants to produce beneficial health effects may result from the fact that the antioxidants can produce harmful effects in some situations, like in established cancer, when sufficient amount of ROS is required to induce apoptosis of malignant cells as discussed above [40, 41]. A third explanation is that the lack of an appropriate method of quantification of redox status has made many clinical trials inconclusive, which does not mean the ineffectiveness of the antioxidants [77]. In fact, the method of quantification of redox status in humans is far from perfect, and many times in clinical trials the redox status had not been measured before starting and after the end of antioxidant therapy [13, 14, 77]. Of note, measuring one or several pro- and antioxidant markers may not provide a comprehensive measure of redox status of the target organ or tissue, and the systemic redox status may not represent the status of the target.

Based on the interdependent nature of oxidative stress and inflammation discussed in this review a new explanation of antioxidant paradox may be proposed. Selection of antioxidants that do not simultaneously inhibit both oxidative stress and inflammation or use of nonselective agents that block some of the oxidative and/or inflammatory pathways but exaggerate the others might be responsible for the failures of the antioxidant clinical trials. To establish the validity of this explanation it would be essential to quantify both redox and inflammatory status before, during, and after the antioxidant therapy. However, an experimental evidence of why a nonselective antioxidant that acts by inhibiting ROS generation and thereby NF-*κ*B activation may fail has been demonstrated in a recent study by Djuric et al. [54]. In a psychosocial stress induced atherosclerotic animal model Djuric et al. showed that selective targeting of NF-*κ*B subunit cRel and maintaining the activity of p50/p65 subunits provided antiatherosclerotic effect by limiting proinflammatory effect of NF-*κ*B without abolishing its anti-inflammatory and antioxidant functions [54]. This finding indicates that a nonselective antioxidant or anti-inflammatory agent that inhibits all the subunits of NF-*κ*B is likely to fail and a selective inhibitor is required to successfully treat the disease. Furthermore, this finding supports the idea that the interdependence between oxidative stress and inflammation may be a reasonable explanation of antioxidant paradox at least in some cases.

## 5. Conclusion

Inflammation and oxidative stress are closely related and tightly linked pathophysiological processes. One of them may appear before or after the other, but when one of them appears the other one is most likely to appear; and then both of them take part in the pathogenesis of many chronic diseases. Although identification and treatment of primary abnormality are of great clinical importance, treating only the primary abnormality may not always be successful, because once the process has been already started, both inflammation and oxidative stress act in concert to accentuate each other and to induce progressive damage. Thus, antioxidant therapy alone is unlikely to prevent diseases known to be induced by oxidative stress, like cardiovascular and diabetic complications, neurodegenerative diseases, cancer, or aging. However, great care should be taken in selection of antioxidant agents, selection of dosage of antioxidants not to produce harmful effects, and, most importantly, quantification of redox and inflammatory status to make appropriate interpretation of the findings.

## Figures and Tables

**Table 1:** Important reactive species in biological system.

Free radicals	Nonradicals
*Reactive oxygen species*	
Superoxide, O_2_^∙−^	Hydrogen peroxide, H_2_O_2_
Hydroxyl, OH^∙^	Singlet oxygen, O_2_^1^Δ*g*
Peroxyl, RO_2_^∙^	Organic peroxides, ROOH
Alkoxyl, RO^∙^	Peroxynitrite, ONOO^−^
Carbonate, CO_3_^∙−^	Peroxynitrous acid, ONOOH
*Reactive chlorine species*	
* *Atomic chlorine, Cl^∙^	Hypochlorous acid, HOCl
	Chlorine gas, Cl_2_
	Nitryl (nitronium) chloride, NO_2_Cl
*Reactive nitrogen species*	
* *Nitric oxide, NO^∙^	Nitrous acid, HNO_2_
* *Nitrogen dioxide, NO_2_^∙^	Nitrosyl cation, NO^+^
	Nitroxyl anion, NO^−^
	Dinitrogen tetroxide, N_2_O_4_
	Dinitrogen trioxide, N_2_O_3_
	Peroxynitrous acid, ONOOH
	Alkyl peroxynitrites, ROONO
